# PREDIVAC: CD4+ T-cell epitope prediction for vaccine design that covers 95% of HLA class II DR protein diversity

**DOI:** 10.1186/1471-2105-14-52

**Published:** 2013-02-14

**Authors:** Patricio Oyarzún, Jonathan J Ellis, Mikael Bodén, Boštjan Kobe

**Affiliations:** 1 School of Chemistry and Molecular Biosciences, Institute for Molecular Bioscience and Australian Infectious Diseases Research Centre, University of Queensland, Brisbane, QLD, 4072, Australia; 2 Biotechnology Centre, Universidad San Sebastian, Bellavista 7, Recoleta, Santiago, 8420524, Chile; 3 School of Information Technology and Electrical Engineering, University of Queensland, Queensland, 4072, Australia

**Keywords:** CD4+ T-cell epitope prediction, Epitope-based vaccination, Immunodominance, MHC (HLA) class II proteins, MHC (HLA) class II polymorphism, Pan-specific, Peptide binding prediction, Peptide vaccination, Specificity-determining residues

## Abstract

**Background:**

CD4+ T-cell epitopes play a crucial role in eliciting vigorous protective immune responses during peptide (epitope)-based vaccination. The prediction of these epitopes focuses on the peptide binding process by MHC class II proteins. The ability to account for MHC class II polymorphism is critical for epitope-based vaccine design tools, as different allelic variants can have different peptide repertoires. In addition, the specificity of CD4+ T-cells is often directed to a very limited set of immunodominant peptides in pathogen proteins. The ability to predict what epitopes are most likely to dominate an immune response remains a challenge.

**Results:**

We developed the computational tool Predivac to predict CD4+ T-cell epitopes. Predivac can make predictions for 95% of all MHC class II protein variants (allotypes), a substantial advance over other available methods. Predivac bases its prediction on the concept of specificity-determining residues. The performance of the method was assessed both for high-affinity HLA class II peptide binding and CD4+ T-cell epitope prediction. In terms of epitope prediction, Predivac outperformed three available pan-specific approaches (delivering the highest specificity). A central finding was the high accuracy delivered by the method in the identification of immunodominant and promiscuous CD4+ T-cell epitopes, which play an essential role in epitope-based vaccine design.

**Conclusions:**

The comprehensive HLA class II allele coverage along with the high specificity in identifying immunodominant CD4+ T-cell epitopes makes Predivac a valuable tool to aid epitope-based vaccine design in the context of a genetically heterogeneous human population.The tool is available at: http://predivac.biosci.uq.edu.au/.

## Background

Epitope-based vaccines (EVs) make use of short, antigen-derived peptides (corresponding to epitopes) that are administered to trigger a protective humoral (B-cell epitopes) and/or cellular (T-cell epitopes) immune response. T-cell epitopes are presented to T-cells in association with major histocompatibility complex (MHC) proteins. While cytotoxic T-cells recognize intracellular peptides displayed by MHC class I molecules (CD8+ T-cell epitopes), T helper cells recognize peptides that are taken up from the extracellular space and displayed by MHC class II molecules (CD4+ T-cell epitopes). The peptide:MHC complex (pMHC) interacts with the T-cell receptor, leading to its activation and subsequent induction of a cellular immune response. EVs offer several potential benefits over traditional vaccines, including the precise control over the immune response activation, the ability to focus on the most relevant antigen regions (conserved and/or highly immunogenic), as well as production and biosafety advantages due to their chemically simple and well-characterized composition. CD4+ T-cell epitopes play a key role in EV design [1], as the cognate help provided by these cells is essential for the generation of vigorous humoral and cytotoxic CD8+ T-cell responses [2]. Because experimental screening of large sets of peptides is time-consuming and costly, *in silico* methods that facilitate CD4+ T-cell epitope mapping on protein antigens are paramount for EV development.

Human MHC class II (HLA class II) proteins are made up of two transmembrane chains: α-chain (34 kDa) and β-chain (29 kDa), which together shape the peptide-binding groove. This region defines five pockets that mostly interact with individual residues of the peptide [3]. The HLA genes are the most polymorphic in the human genome. Currently, the IMGT/HLA database [4] lists 1679 HLA class II alleles associated with three classical loci (1267 DR, 223 DQ and 189 DP alleles), corresponding to 931 distinct HLA class II DR protein variants (allotypes; as of September 2012), and this number continues to grow at a rate of 200 alleles per year [5]. This huge diversity poses serious problems for vaccine design, as different alleles are expressed at dramatically different frequencies in different ethnicities. Individuals display different sets of alleles that likely respond to a different set of peptides from a given pathogen.

The MHC class II binding groove is open at both ends, allowing peptides (~9-22 residues long) to project out of the groove, causing ambiguity in their positional alignment and making the alignment a fundamental step in predicting binding. Two classes of methods for CD4+ T-cell epitope prediction have emerged. The first category (data-driven methods) relies on peptide sequence comparisons to identify binding motifs, and includes the pioneer method SYFPEITHI [6] and matrix-based approaches such as position-specific binding profiles (e.g. Rankpep [7], ARB [8] and SMM-align [9]). TEPITOPE [10] and TEPITOPEpan [11] are based on the so-called “pocket profiles” and MultiRTA [12] is based on thermodynamic principles. Another group of data-driven approaches involves machine learning, which has been proposed to capture subtle MHC class II-binding patterns (e.g. NN-align [13], NetMHCIIPan-2.0 [14] and MHCIIMulti [15]). The second category involves structure-based methods. These methods perform molecular modeling calculations on pMHCs in order to estimate the binding energies, thus offering independence from experimental binding data. A recent analysis showed them to be better than random, but inferior to state-of-the-art data-driven approaches [16].

The few methods able to cope with the extent of HLA class II polymorphism are collectively referred to as pan-specific approaches [17]. Although these methods (NetMHCIIPan-2.0, TEPITOPEpan, MultiRTA) are potentially suitable for EV design, they do not fully account for the entire allotypic diversity of human ethnic populations and they do not take into consideration immunodominance. Our new tool Predivac described in this paper implements a different pan-specific approach based on the concept of specificity-determining residues (SDRs). This methodology has been previously described by our group for the prediction of substrate specificity of protein kinases [18-20]. While the binding interface of a protein can be extensive, only a small group of SDRs is responsible for specific interactions. The SDRs have been mainly studied in peptide recognition domains (e.g., PDZ, SH3 and kinase domains) of proteins with roles in signalling pathways [21]. Identifying mutations that alter specificity may require a large amount of experimental work; therefore, a number of computational approaches have been developed to predict SDRs. Most of these methods are based on multiple sequence alignments and the use of statistical analysis and evolutionary information to identify SDRs [22]. Other approaches combine multiple sequence alignments with structural information of the binding site [23,24] or physical properties [25]. Like other bioinformatics methods for CD4+ T-cell epitope prediction, Predivac focuses on predicting pMHC complex formation. The method assumes that T cells with the required specificity will be present in the T-cell repertoire. However, despite improvements in the performance of methods predicting MHC class II peptide binding, a recent study showed that state-of-the-art methods are still unsuccessful in predicting CD4+ T-cell epitopes [26], highlighting the need to develop new approaches that cope better with epitope discovery. A significant source of complexity in EV design comes from the fact that most of the response is mounted against a few so-called immunodominant epitopes, despite the presence of many potential epitopes within an immunogen. This restricted antigenic specificity of T cells poses a serious challenge for EV design, as vaccine formulations built on epitopes that do not dominate the immune response will not induce effective protection in the vaccinated organism. Recent evidence suggests that the pMHC kinetic stability plays a central role in controlling MHC class II peptide immunogenicity [27]. In concordance with this model, a strong correlation has been observed between high affinity HLA class II peptide binding, immunodominance and promiscuous CD4+ T-cell recognition [28]. Several studies support this correlation both for MHC class I [29] and class II proteins [30]. Predivac was consequently developed using high-affinity binding data, on the assumption that it is the positive bias toward capturing underlying peptide features that correlates with promiscuity and immunodominance, two properties that are fundamental for EV design [31].

In this study, we introduce the pan-specific method Predivac for CD4+ T-cell epitope prediction, which is based on the SDR concept previously applied to protein phosphorylation site prediction. We assessed the performance of Predivac by cross-validation and compared the predictive performance against several state-of-the-art methods in terms of HLA class II peptide binding and CD4+ T-cell epitope prediction. The comparisons showed that Predivac performed comparable to ten competing methods in high-affinity binding prediction, but delivered the highest specificity in CD4+ T-cell epitope identification, with a particularly strong performance in immunodominant epitope identification, compared to three other pan-specific approaches.

## Methods

### Software implementation

Predivac is written in Perl (v.5.10). Its main component is a purpose-built database (PredivacDB) constructed using Berkeley DB. The web implementation available to the research community is written in Python/CGI. The server operates on GNU/Linux 2.6 and runs Apache 2.2.16. The software performs three major tasks: i) SDR identification; ii) binding data retrieval (from PredivacDB database) and iii) peptide binding prediction.

### SDR identification

HLA class II amino acids involved in peptide ligand recognition were identified using a dataset of 37 peptide:HLA class II complex structures, accounting for 10 different alleles (DR and DQ loci; Table 1). For each binding position, residues within a van der Waals radius of 4 Å from the peptide amino acid were considered as contacting positions (Pymol, DeLano Scientific). Structural alignments of pMHCs were performed with VMD [32]. Sequence variability of HLA molecules at the binding interface was mapped onto the DRB1*0101 allele structure [PDB:1A6A] using the Protein Variability Server (PVS) [33]. SDR identification was also aided by a previous quantum chemistry-based analysis of peptide:HLA class II interaction [34], where the variation of electrostatic properties associated with each of the residues involved in peptide binding was assessed and amino acids inducing a global variation of electrostatic properties in the binding groove (anchoring amino acids) and those involved in specific electrostatic variations (recognition amino acids, responsible for a differential effect) were identified.

**Table 1:** HLA class II crystal structures employed to identify SDRs

**Allele**	**Peptide sequence**	**PDB IDs**
DRB1*0101	AA**YSDQATPLL**LS	1T5X; 1T5W
	AG**FKGEQGPKG**EPG	2FSE
	PE**VIPMFSALS**EGAT	1SJE
	PE**VIPMFSALS**EG	1SJH
	GEL**IGILNAAKV**PAD	1KLG; 2IAM
	GEL**IGTLNAAKV**PAD	1KLU; 2IAN
	GSD**WRFLRGYHQ**YA	1AQD
	PK**YVKQNTLKL**AT	1DLH; 1FYT; 1HXY; 1JWM; 1JWS; 1JWU; 1KG0; 1LO5; 2G9H; 2ICW; 2OJE; 1R5I
DRB1*0301	PVSK**MRMATPLLM**QA	1A6A
DRB1*0401	AY**MRADAAAGG**A	2SEB
	PK**YVKQNTLKL**AT	1J8H
DRB1*1501	ENPV**VHFFKNIVT**P	1BX2; 1YMM
	MDFAR**VHFISALHG**LGGG	2WBJ
DRB3*0101	A**WRSDEALPL**G	2Q6W
DRB3*0301	QV**IILNHPGQI**SA	3C5J
DRB5*0101	GGV**YHFVKKHVH**ES	1H15
	NPVVHF**FKNIVTPRT**PPPSQ	1FV1
	VHF**FKNIVTPRT**PG	1ZGL
DQB1*0201	LQ**PFPQPELPY**	1S9V
DQB1*0602	MN**LPSTKVSWA**AVGGGGSLV	1UVQ
DQB1*0302	SG**EGSFQPSQE**NP	2NNA
	LV**EALYLVCGE**RGG	1JK8

The binding cores of the peptide ligands are highlighted in bold. Underlined PDB IDs represent the structures that were employed for structural alignment.

### PredivacDB database

A key part of the method is PredivacDB, a purpose-built database of nonameric high-affinity binding peptides and SDRs. The data in PredivacDB was gathered from three online repositories (i) The Immune Epitope Data Base (IEDB) [35]; (ii) MHCBN [36] and (iii) EPIMHC [37]. The collected peptides were additionally filtered by eliminating: (i) sequences with length < 9 residues; (ii) non-natural peptides and sequences having Ala percentage >50%; (iii) sequences containing a non-natural atom or group; and (iv) peptides whose binding affinity was determined only through whole-cell based assays. The EasyGibbs method [38], based on the Gibbs sampler approach, was employed to identify nonameric binding cores, as it is a well-validated tool for motif retrieval in MHC class II ligands. For each set of peptides, the retrieved scoring matrix was employed to identify the binding regions in the peptides. For datasets with small numbers of peptides, binding motifs were obtained using the MHC Motif Viewer [39]. PredivacDB was built using the identified nonameric regions and contains 2695 high-affinity sequences accounting for 29 HLA class II alleles (Additional file 1: Table S1).

### Peptide binding prediction

Predivac predicts peptide binding by establishing a correlation between the SDRs in the HLA query protein and the SDRs associated with HLA proteins of known specificity. The process involves the following steps: (i) SDRs for each of the five binding positions are identified in the query HLA protein sequence; (ii) PredivacDB is queried and amino acid frequencies and weights are calculated (Equation 1) for peptide sequences associated with allotypes sharing similar SDRs as the query protein at each binding position; and (iii) a position-weight matrix (PWM) is built based on the binding data, consisting of 20 columns (amino acids) × 5 rows (binding sites).

(1) $$w_{i,j}=\log\left(p_{a,i}/p_{a}\right)$$

Where *p*_*a,i*_ is the probability of observing amino acid *a* at position *i* of the peptide (*i*= 0,…,8), and *p*_*a*_ is the probability of observing amino acid *a* in the background model. The frequency of a residue at position *i* in the peptide (*f*_*a,i*_) is estimated using pseudocounts by adding $\sqrt{n}$/20 to the raw frequency *f*_*a,i*_ and dividing by *n+*$\sqrt{n}$, were n is the number of sequences used to calculate the frequency. SDRs are considered similar if substitution using the BLOSUM62 matrix gives a positive score.

Finally, T-cell epitope mapping is carried out by parsing query protein sequences into overlapping nonameric segments (peptides), each of which is assigned a binding score using the PWM (sliding window technique). Raw scores are normalized to a 1-100 range using a linear transformation, considering the minimal and maximal theoretical peptide scores that can be obtained from the PWM. A sensible cutoff to discriminate peptides that bind from those that do not is 60.

## Results and discussion

### Specificity-determining residues

**Figure 1:**
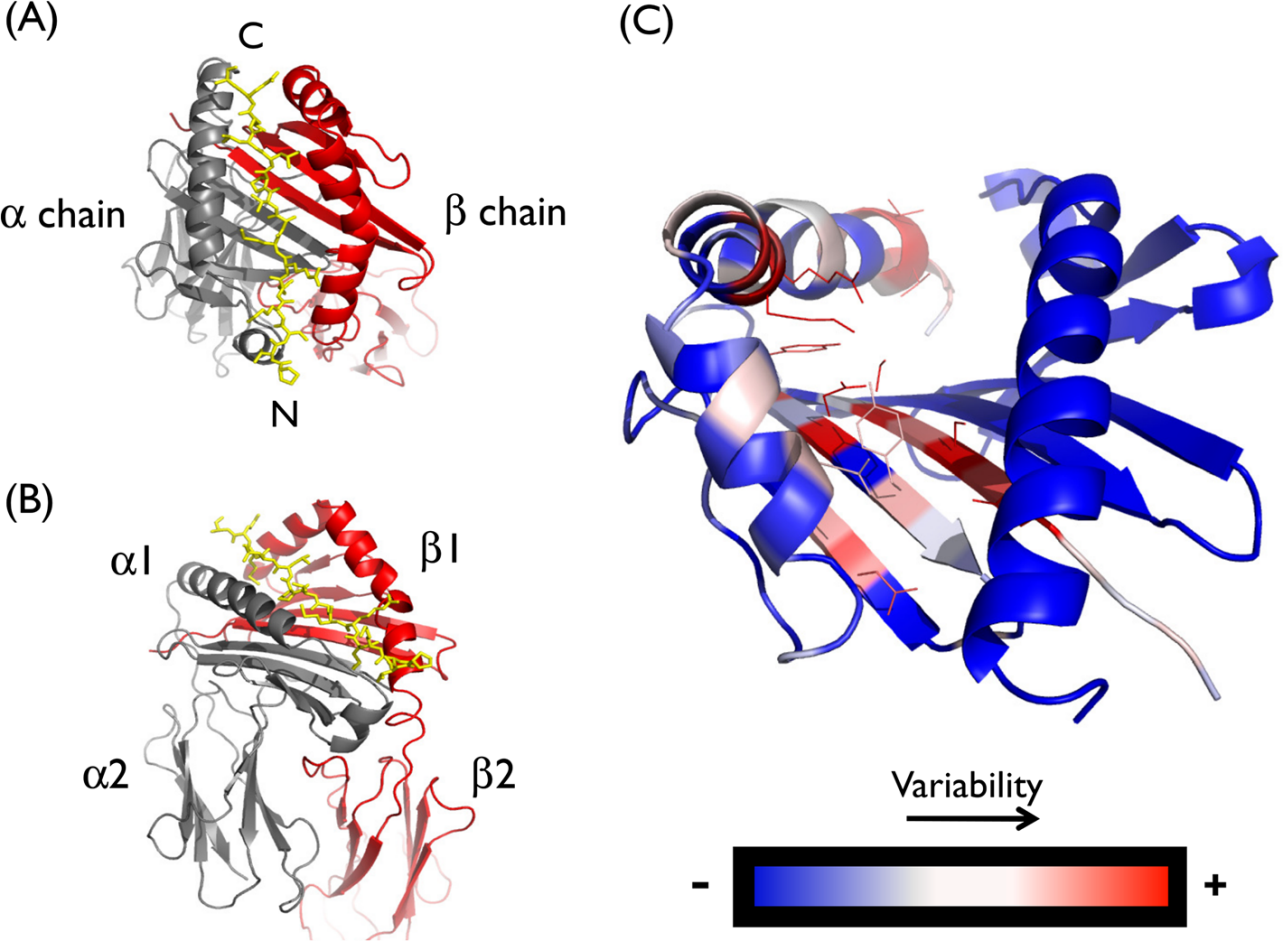
**HLA class II polymorphism and three-dimensional structure.** [PDB:1A6A] is used as a representative structure in this figure. **(A)** Ribbon diagram of the binding groove. Chain α, grey; chain β, red. Each chain consists of two domains (α1, α2 and β1, β2, respectively). Only the ectodomains α1 and β1 shape the binding groove. **(B)**, as (a), but showing the entire molecule. **(C)** Three-dimensional representation of the sequence variability in the binding groove, highlighting the location of SDRs in the β1 domain. Variability is represented in a two-colour scale from blue to red, where blue indicates non-variable positions and red indicates variable positions.

Structural alignments of the binding groove (α- and β-domains) were performed with 17 distinct pMHCs out of 37 structures analyzed (Table 1), accounting for 7 different DR alleles. In cases where a particular complex is represented by more than one structure, the highest resolution one was selected. The root-mean-square-distance of the residues constituting the binding groove was 0.45 Å, considering Cα atoms of both domains, reflecting the high level of structural conservation of residues dictating specificity. This is a condition required for the SDR method to be applicable. As shown in Figure 1, the β-domain accounts for the specificity of the HLA class II proteins, given that 99% of polymorphisms are present exclusively in this domain [40]. On the other hand, there are significantly less polymorphic positions in alleles belonging to the DP and DQ loci, and these are distributed both through the α- and β-domains of the binding groove [40]. The application of the method for these alleles must be assessed further, as the specificity seems to be determined by a larger number of residues than in the case of DR alleles. In addition, limited binding data is available for HLA DP and DQ molecules.

Fifteen contacting positions on the polymorphic β-domain were finally considered as SDRs, providing they exerted an influence on peptide binding, they were structurally conserved, they were polymorphic and they were identified previously [34] to have an influence on the electrostatic properties of the binding groove (Additional file 1: Figure S1). These key positions allow the relative weight of the effect of all amino acids on each binding site to be determined, and predicting whether a peptide is a likely ligand for a given HLA class II protein. It is worth noting that, whereas in Predivac the weights are calculated from binding data (retrieved from PredivacDB), the popular method TEPITOPE does this using experimentally determined binding affinities (referred as “pocket profiles”).

### Performance assessment and cross-validation

Predivac was assessed both in terms of HLA class II binding prediction and CD4+ T-cell epitope prediction, including using a sub-set consisting exclusively of immunodominant epitopes. The accuracy of the predictions was measured in terms of the area under the ROC curve (AUC), which represents the probability that the classifier will assign a higher score to a positive example compared to a negative example.

For consistency with previous studies [14,41], Predivac’s accuracy on HLA class II alleles from PredivacDB was assessed using a modification of the leave-one-out cross-validation methodology, whose objective is to minimize the similarity between the training and test datasets. This testing procedure, called leave-one-(allele)-out cross-validation, involves the exclusion of a single allele from the database and then assessing the performance using the binding data associated with that particular excluded allele. Balanced datasets were constructed for AUC calculation, to have equal number of high-affinity binders (positives) and non-binders (negatives). The calculation was repeated ten times using different non-binder datasets chosen randomly from the ligand source.

For every tested allele, both for peptide binding and CD4+ T-cell epitope prediction, positive datasets were constructed using the whole set of epitopes restricted by it. The negative datasets were constructed by splitting the protein sequences into overlapping peptides of the same length as the particular epitope and all peptides except the annotated peptide were taken as negatives. This is an established validation strategy, based on the stringent assumption that the misclassification of a few potential peptides (epitopes, representing false negatives) can only lead to a slight decrease in the overall predictive performance of the prediction. Redundancy in PredivacDB was removed by excluding all nonameric peptides equal or containing any of the sequences present in the CD4+ T-cell epitope datasets.

### HLA class II peptide binding prediction

**Figure 2:**
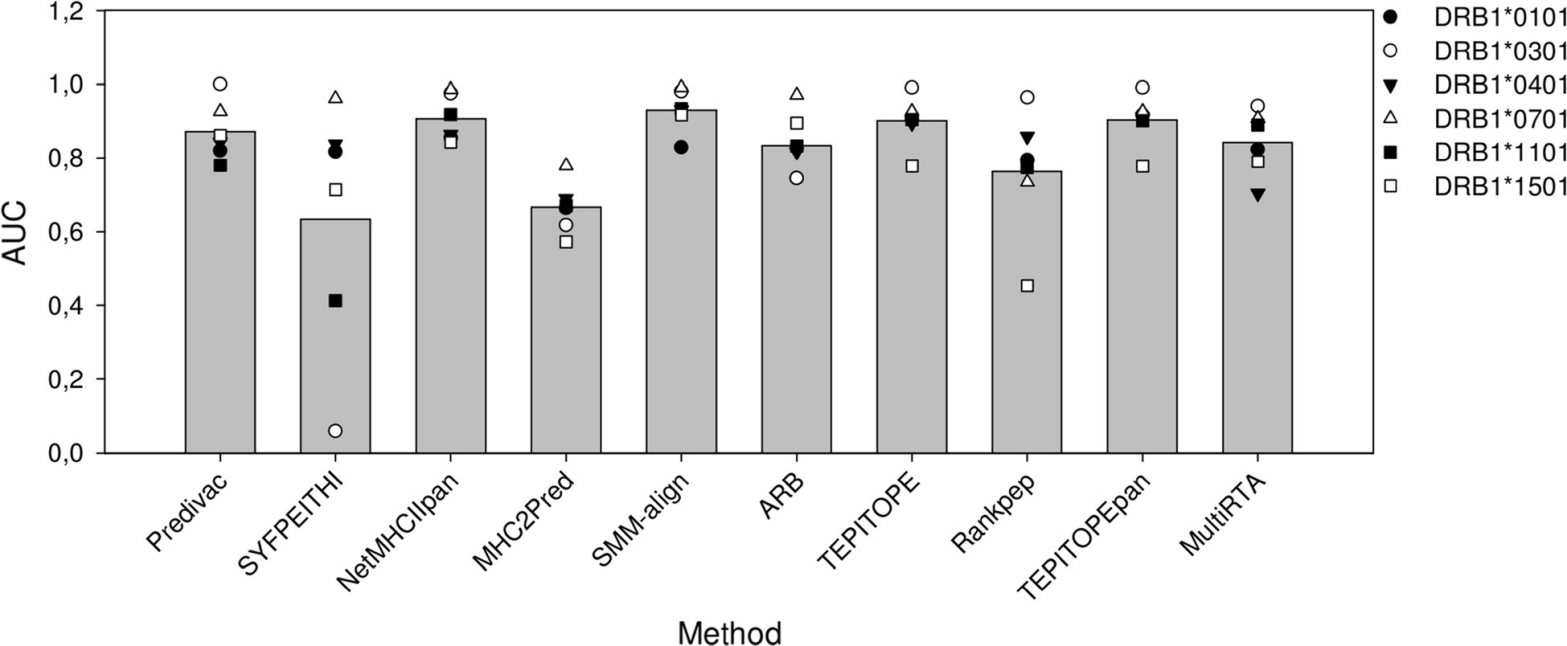
**Comparison of ten web-accessible methods for HLA class II peptide-binding prediction.** Only peptides displaying high-affinity binding (IC50 ≤ 50 nM) from the DFRMLI dataset were used. Bars indicate the average AUCs.

Predivac implements a method to predict CD4+ T-cell epitopes that allows coverage of 883 out of 931 HLA class II DR locus proteins, i.e., 95% of human protein diversity. Other pan-specific approaches account for 700 (TEPITOPEpan), 500 (NetMHCIIPan-2.0) and 430 (MultiRTA) HLA class II allotypes (according to data reported in the original publications and web-sites). Additional file 1: Figure S2 illustrates the performance of Predivac on the prediction of peptide binding to HLA class II alleles different from those contained in PredivacDB. The performance of the method was evaluated for 14 alleles with binding data for at least 25 peptides each. The average AUC was 0.842. In addition, the performance of Predivac was tested and compared with nine state-of-the-art methods implemented as web-servers using the DFRMLI dataset [42], which has been previously employed for benchmarking purposes [43]. It represents the binding affinity distribution in full-length protein antigens, comprising 103 MHC class II peptides (15 to 19 residues long) derived from four protein antigens. The binding data are highly standardized, accounting for seven HLA-DR molecules (DRB1*0101, DRB1*0301, DRB1*0401, DRB1*0701, DRB1*1101, DRB1*1301 and DRB1*1501). In this study, binding peptides were defined as those with IC_50_ ≤ 50 nM. For DRB1*0701, the threshold was set at 100 nM, while DRB1*1301 was left out because it does not contain high-affinity binders. Additional file 1: Table S2 lists the methods compared. Predivac performed comparably to state-of-the-art methods. The highest average AUC was delivered by SMM-align (0.930), with 0.872 for Predivac (Figure 2).

### CD4+ T-cell epitope identification

**Figure 3:**
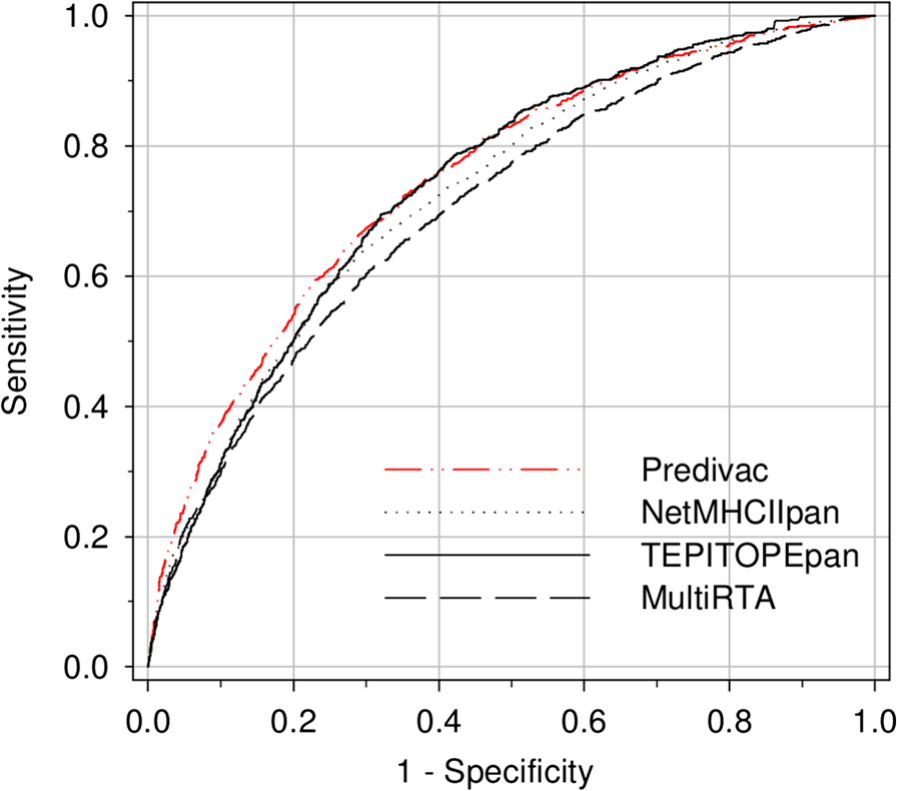
**Comparison of overall predictive performance of four pan-specific methods.** Predivac, NetMHCIIPan-2.0, TEPITOPEpan and MultiRTA were compared in identifying CD4+ T-cell epitopes.

**Figure 4:**
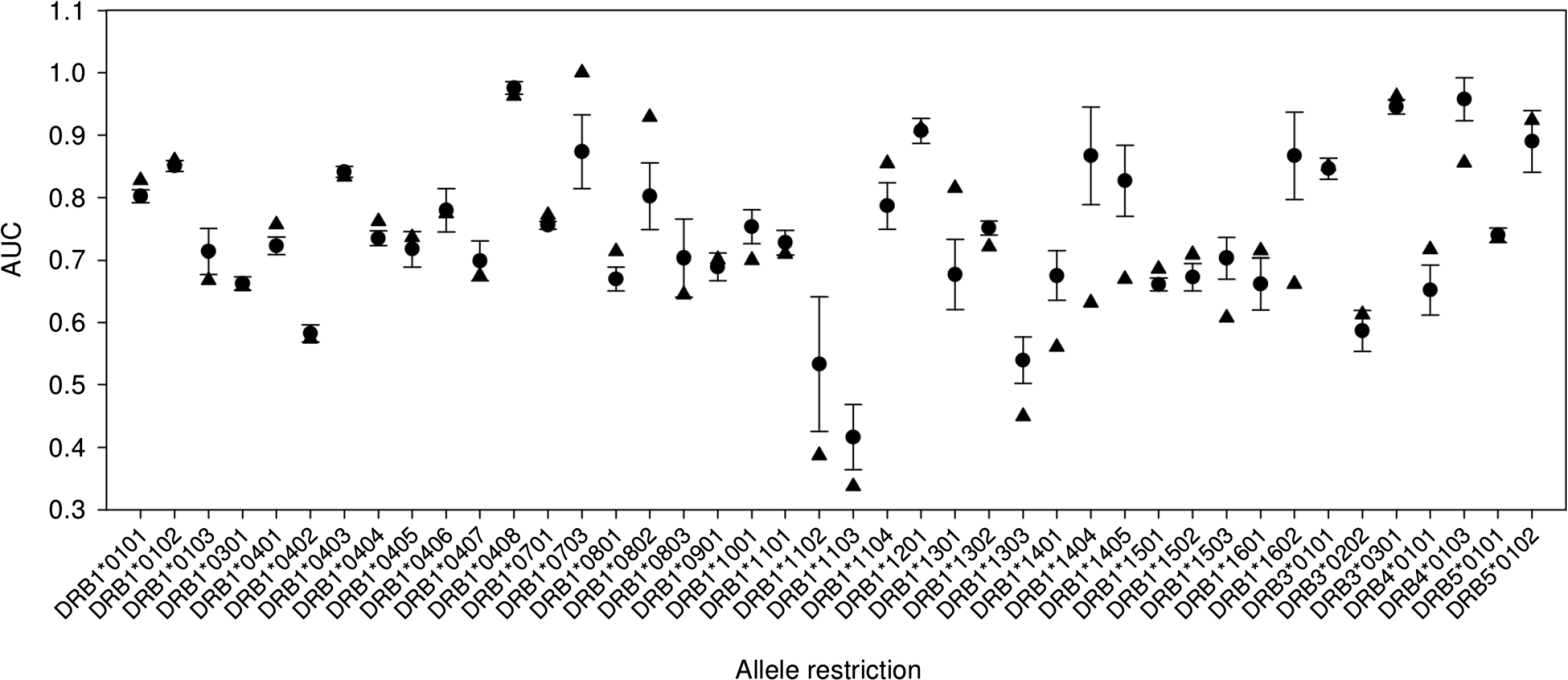
**Comparison of the predictive performance of Predivac and the average performance of four pan-specific methods.** Predivac (triangles) and the average performances (circles) of NetMHCIIPan-2.0, TEPITOPEpan and MultiRTA were compared in identifying CD4+ T-cell epitopes for 43 HLA class II alleles in the dataset. Bars indicate the standard error

The capability of Predivac to identify CD4+ T-cell epitopes was assessed using a dataset gathered from IEDB, consisting of 1325 of MHC class II-restricted T-cell epitopes that account for 43 HLA class II alleles (http://www.cbs.dtu.dk/suppl/immunology/NetMHCIIpan-2.0/). Only the pan-specific approaches Predivac, NetMHCIIPan-2.0, TEPITOPEpan and MultiRTA were considered for performance comparison, as they afford prediction for the whole set of alleles restricting those epitopes (MHCIIMulti website was not available). As shown in Figure 3, the resulting AUCs were (in decreasing order) 0.749 (Predivac), 0.741 (TEPITOPEpan), 0.728 (NetMHCIIPan-2.0) and 0.710 (MultiRTA). Predivac ROC curve showed a slight dominance over the high-specificity region (highest true positive/false positive ratio), which means that the method delivers the highest performance in identifying immunodominant epitopes correctly. This ability is particularly relevant given the skewed distribution of the dataset, where only one peptide per protein is positive (the epitope) and all the remaining peptides are negatives. When the performance was evaluated for each individual allele (Additional file 1: Table S3), average AUCs of 0.760 (TEPITOPEpan), 0.759 (NetMHCIIPan-2.0), 0.730 (Predivac) and 0.725 (MultiRTA) are obtained. These average AUCs differ slightly from those previously published values using the same dataset [11], which is a consequence of differences in the calculation methodology. In our study, AUC values were calculated using one dataset per allele or per method, containing simultaneously all positive (epitopes) and negative examples (as described above), while in the referred study the AUC values were individually calculated for each protein and subsequently averaged to obtain an allele’s performance (all protein AUCs associated with each allele) and a method’s performance (all allele AUCs). Our approach to calculate unique AUC values over the entire dataset offers potentially a better representation of the overall performances. This is further supported by the unbalance in the dataset, which means that certain alleles that are considerably underrepresented (e.g., six alleles are accounted by only one epitope/protein) will have the same weight as DRB1*0401 (342 examples) if using the averages. In particular, Predivac’s average is significantly affected by its poor performance on two alleles, DRB1*1602 and DRB1*1404, having 3 and 1 epitopes, respectively. Figure 4 shows the predictive performance of Predivac for every tested allele in relation to the average performance of all the pan-specific methods. The low standard errors demonstrate a degree of association among the predictive performances over each particular allele, likely reflecting limitations in the ability of predicting CD4+ T-cell epitopes common to all algorithms. The large gap between the performance of the methods in predicting high-affinity peptide binding (AUC_max_ ~0.9) and CD4+ T-cell epitopes (AUC_max_ ~0.75) also supports the hypothesis that binding affinity is not the only factor governing CD4+ T-cell epitopes presentation. Abundant evidence points to the influence of local structural properties of the antigen in the processing and presentation of the epitopes [44].

An additional benchmarking dataset, based on influenza virus-specific CD4+ T-cell epitopes from five major influenza virus proteins in mice expressing a distinct set of class II molecules, was obtained from a recently published study [26]. For DR1-restricted epitopes (Additional file 1: Figure S3), both Predivac and NetMHCIIPan-2.0 reached a comparable accuracy (AUC 0.700), although Predivac delivered the highest specificity. For DR4-restricted epitopes (Additional file 1: Figure S4), Predivac outperformed the rest of the methods with an AUC of 0.743, followed by NetMHCIIPan-2.0 (AUC 0.696), TEPITOPEpan (AUC 0.685) and MultiRTA (AUC 0.641).

### High-affinity binding and immunodominance

Available evidence suggests that CD4+ T-cell immunodominance correlates best with the dissociation rate constant *k*_*off*_[45], and dominant epitopes have been associated with half-lives (*t*_*1/2*_) ≥ 100 h [46]. A strong correlation has been established between pMHC class II high-affinity binding, immunodominance and promiscuity, with half-maximal inhibitory concentration (IC_50_) values typically fluctuating around a few nM [47], justifying our affinity threshold of 50 nM for peptide selection in PredivacDB. We set this threshold in order to drive the specificity prediction exclusively by high-affinity binding data and thus potentially favour the identification of immunodominant CD4+ T-cell epitopes.

The dissociation rate constant (*k*_*off*_) plays a crucial role in ligand-receptor interactions, as it varies over many orders of magnitude, while the association rate constant (*k*_*on*_) is often controlled by the rate of diffusion [48]. This observation is supported by the on-rates for pMHC class II complexes in the AntiJen database [49] (Additional file 1: Table S4), and the kinetics of twenty different peptides binding to the MHC class II protein, showing diffusion-limited *k*_*on*_ values of 10^4^-10^5^ M^-1^s^-1^ and *k*_*off*_ values spanning a range >10^4^-fold [50]. A half-life (*t*_*1/2*_) of 100 h is equivalent to a *k*_*off*_ value of 1.925 × 10^-6^ s^-1^ (*k*_*off*_ = 0.693/*t*_*1/2*_); thus, the dissociation constant (*K*_*d*_ = *k*_*off*_/ *k*_*on*_) would be between ~0.02 nM (*k*_*on*_ = 10^5^ M^-1^s^-1^) and ~0.2 nM (*k*_*on*_ = 10^4^ M^-1^s^-1^) for the high-affinity interaction range usually associated with CD4+ T-cell immunodominance.

### Immunodominant epitope prediction

Immunodominant and promiscuous epitopes are the best candidates for EV design; therefore, we benchmarked Predivac in terms of its ability to identify immunodominant epitopes in full-length proteins. Immunodominance is an allele-restricted property that in the context of one individual refers to those epitopes that elicit the largest immune response. However, because many immunodominant CD4+ T-cell epitopes have the ability to bind with high-affinity and to be permissively presented in the context of multiple DR molecules [51], this study focused on CD4+ T-cell epitopes whose response has been examined in the context of many individuals expressing diverse haplotypes. In this case, immunodominant epitopes are those that elicit the most common response, which may or may not be immunodominant within the hierarchy of responses of particular individuals.

A dataset containing 42 immunodominant CD4+ T-cell epitopes with known allele restriction (12 different HLA class II alleles) was gathered from the literature (Additional file 1: Table S5 and Additional file 2), using a previously published dataset [52] as a basis. The dataset contains nonameric epitopes known as “universal epitopes”, as they bind in an apparently indiscriminate manner to most DR alleles, such as those contained in the bacterial tetanus toxoid protein (epitope Nº3 in Additional file 1: Table S5) and in the hemagglutinin influenza virus protein (epitope Nº1). It has been suggested that these peptides bind the MHC molecule in the same register but with interactions driving T-cell recognition variably influenced by the regions flanking the core [53]. Moreover, longer epitopes are often clusters of dense antigenic regions (hot-spots), such as the immunodominant epitope spanning residues 378 to 398 of the circumsporozoite protein sequence (epitope N°36-41), which can bind with several overlapping 9-mer registers within its 20-mer sequence.

Only minimal immunogenic regions (MHC class II peptides) with lengths ≤ 13 amino acids were considered in the dataset, corresponding to the shortest fragment able to elicit a significant proliferative CD4+ T-cell response. DRB1*0101 was kept as a reference allele for all promiscuous epitopes sharing this restriction. AUC values were obtained for each allele and for the full dataset. In addition, for the dataset of immunodominant epitopes, the ranking corresponding to the epitope score in a sorted list of all the scores of same-length peptides in the source protein was recorded as a percentage (epitope score ranking/total number of peptides). The ranking position corresponds to the highest position of a predicted binding core/epitope (9-mers) where a full-length match occurs with the actual immunodominant epitope (9- to 12-mers). For methods hosted at the IEDB website (SMM-align, TEPITOPE and ARB), which retrieve binding predictions over 15-mer sequences, only the nonameric core associated with the optimal 15-mer sequence was considered.

**Figure 5:**
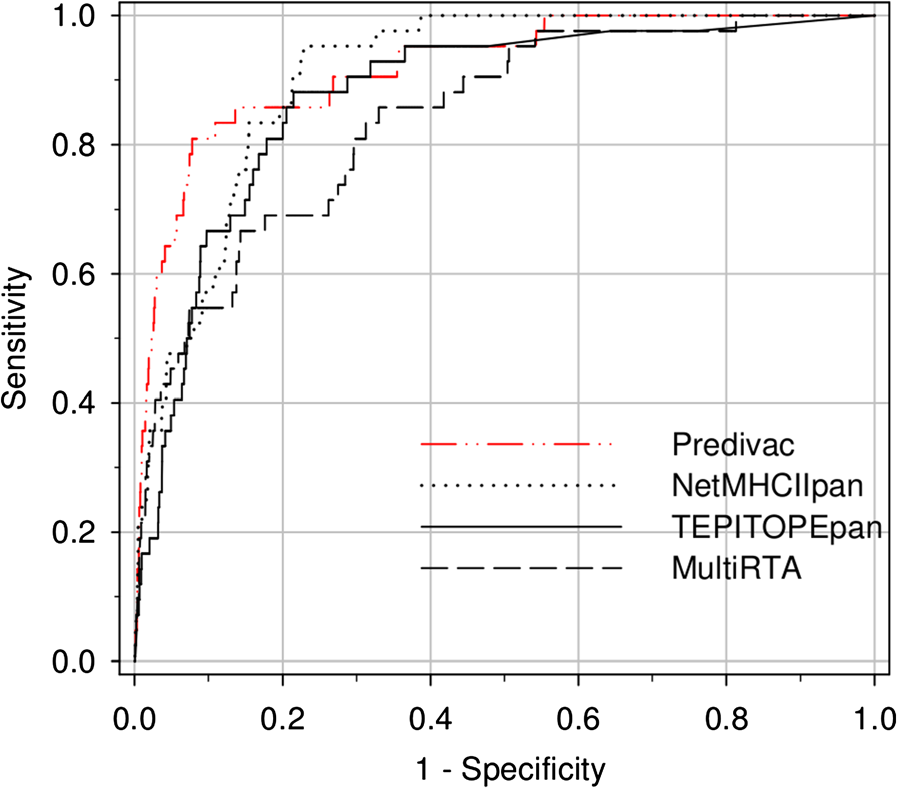
**Overall predictive performance of of four pan-specific methods in identifying immunodominant CD4+ T-cell epitopes.** Overall predictive performance of Predivac, NetMHCIIPan-2.0, TEPITOPEpan and MultiRTA in identifying immunodominant CD4+ T-cell epitopes is compared.

**Figure 6:**
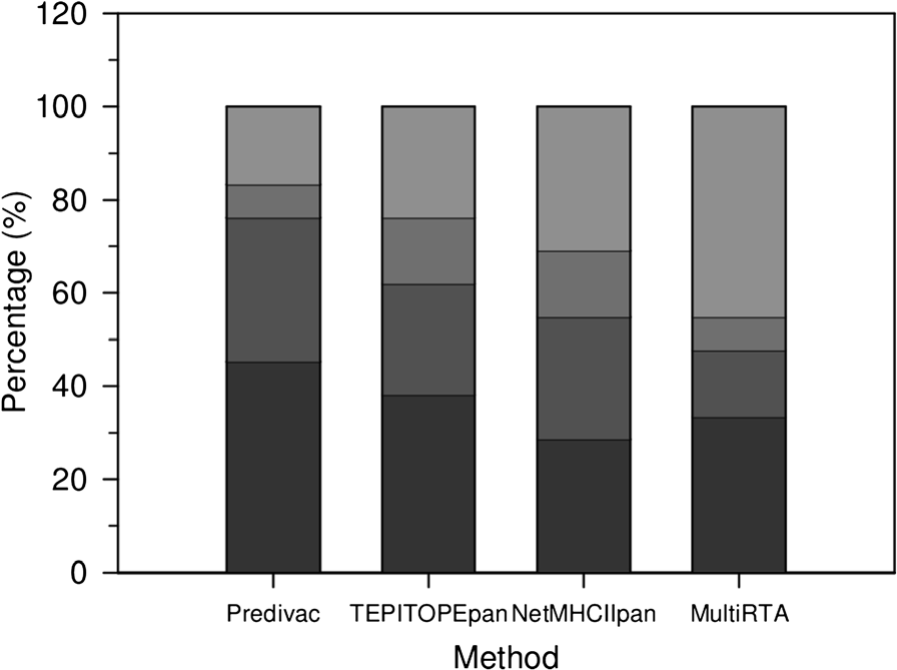
**Distribution of the percentages corresponding to the rankings associated with all the CD4+ T-cell immunodominant epitopes in the context of their corresponding source proteins, for four pan-specific methods.** Predivac, TEPITOPEpan, NetMHCIIPan-2.0 and MutiRTA are compared. From the bottom up, the first stack represents the percentage of immunodominant epitope scores in 0-1% of top scores; second stack: 1-3%; third stack: 3-5%; forth stack: remaining 5-100%.

Overall, Predivac outperformed other pan-specific methods, with accuracy slightly higher than NetMHCIIpan (AUCs 0.918 (Predivac), 0.907 (NetMHCIIPan-2.0), 0.878 (TEPITOPEpan) and 0.840 (MultiRTA)) (Figure 5). Predivac’s ROC curve for immunodominant CD4+ T-cell epitopes was superior paticularly over the high-specificity region (Additional file 1: Figure S5). Fractional AUC values for the curves over the high-specificity region (specificity range 0.8-1.0) were 0.145 (Predivac), 0.117 (NetMHCIIPan-2.0), 0.109 (TEPITOPEpan) and 0.104 (MultiRTA). AUC values are within the same range (around 0.9) as the values obtained for high-affinity binding prediction (Figure 2), supporting the hypothesis that the pMHC complex stability is key in dictating CD4+ T-cell immunodominance, perhaps overpowering other factors, such as local structural properties, that would play a role in subdominant epitopes. The average rankings associated with the immunodominant epitopes in their source proteins were 2.75 ± 4.39 (Predivac), 5.40 ± 8.09 (NetMHCIIPan-2.0), 8.18 ± 13.30 (MultiRTA) and 8.71 ± 22.10 (TEPITOPEpan). Figure 6 presents the distribution of these rankings, showing that Predivac outperformed other approaches by identifying 45% of immunodominant epitopes within the top 1%, and 75% of the epitopes within the top 3% of the scores (the details for each epitope/method are shown in Additional file 1: Table S6). This result is in agreement with the high specificity demonstrated by Predivac in the AUC analysis. The second-top performance was delivered by TEPITOPEpan, in agreement with the fact that this method is a pan-specific extension of the original high-quality experimental binding data driving the TEPITOPE method (pocket profiles). TEPITOPE has been able to identify more than 75% of immunodominant HLA class II endogenous ligands for 1–6% top-scoring peptides [10], which is consistent with the range for our dataset of exogenous immunodominant epitopes. It is expected that TEPITOPEpan inherited these features. Both NetMHCIIPan-2.0 and MultiRTA are trained on datasets containing both high and moderate peptide binders (IC50 ≤ 500 nM), which could explain to some extent the slightly lower performance on immunodominant epitopes.

It is worth noting that CD4+ T-cell epitope predictions for Predivac, NetMHCIIPan-2.0 and MultiRTA are likely to present some bias for those alleles present both in the training datasets and the test sets. Only TEPITOPEpan does not depend on experimental binding data. Even by removing peptide redundancy in PredivacDB, it is to be expected that the remaining similar peptides contribute to improving Predivac’s predictive performance on these alleles. However, the primary objective of this exercise was benchmarking the pan-specific approaches under equivalent conditions, therefore; despite this bias the analysis remains informative.

## Conclusions

The Predivac method introduced in this paper implements a pan-specific approach for HLA class II binding predictions based on the SDR concept. It displays a wider coverage yet performs comparable to other available methods. For CD4+ T-cell epitope prediction, Predivac delivered the highest specificity, which is valuable in epitope discovery, given the need to correctly identify a few CD4+ T-cell epitopes among a large number of non-epitopes. Furthermore, an association with individual allotypes was observed among the AUC values for all methods, which possibly highlights a barrier imposed by biological variables not accounted for by the models. All methods performed better when they were assessed on immunodominant CD4+ T-cell epitopes, reaching similar accuracy levels to those obtained for high-affinity peptide-binding prediction. For the immunodominant CD4+ T-cell epitope dataset, Predivac outperformed competing methods (AUC 0.918), particularly standing out in terms of its high specificity, which allowed identification of 75% of immunodominant epitopes within the top 3% scoring peptides. This outcome supports our thesis that driving the prediction with high-affinity binding data can impose an advantageous bias toward capturing underlying peptide features that correlate with stable peptide-MHC binding, and subsequently increases the probability of identifying immunodominant CD4+ T-cell epitopes.

Predivac users need to be aware of the limitations imposed by the approach Predivac is based on. The method assumes that interactions between the protein and the peptide are independent for each peptide position, and the prediction of CD4+ T-cell epitopes focuses solely on peptide binding to the MHC molecule as the most discriminative determinant of immune response. These limitations will be addressed in future work.

Predivac provides CD4+ T-cell epitope predictions over 95% of HLA class II DR allotypes, making it a valuable tool to aid EV design in the context of an ethnically heterogeneous population. Ultimately, by enabling highly specific immunodominant epitope identification, we expect our tool to be helpful in the vaccination strategy of targeting simultaneously multiple dominant and subdominant epitopes from one or several pathogen proteins, overcoming the propensity of the immune system to focus on a very limited set of epitopes. We are currently extending the tool to better facilitate EV design by considering allele frequency data and population coverage.

## Abbreviations

AUC: Area under the ROC curve; DFRMLI: Dana-farber repository for machine learning in immunology; EV: Epitope-based vaccine; HLA: Human leukocyte antigen; IEDB: Immune epitope data base; LOO-CV: Leave-one-out cross-validation; MHC: Major histocompatibility complex; PWM: Position weight matrix; ROC: Receiver-operator characteristic; SDR: Specificity-determining residue.

## Competing interests

The authors declare that they have no competing interests.

## Authors’ contributions

Designed research: PO, JJE, BK; performed research: PO, JJE; analyzed data and wrote the paper: all authors. All authors read and approved the final manuscript.

## Supplementary Material

Additional file 1 **Supplementary tables and figures.** This pdf file contains **Tables S1-S6** and **Figures S1-S5**. Click here for file

Additional file 2 **Immunodominant epitope dataset.** This text file contains the immunodominant CD4+ T-cell epitope dataset, including their associated allele restrictions and source protein sequences. This dataset was employed to assess the performance of Predivac, NetMHCIIpan, TEPITOPEpan and MultiRTA in identification of these epitopes. Click here for file
