# Supplementary material for: PREDIVAC: CD4+ T-cell epitope prediction for vaccine design that covers 95% of HLA class II DR protein diversity
Source: BMC Bioinformatics. 2013 Feb 14;14:52. doi: 10.1186/1471-2105-14-52 (PMC3598884; doi:10.1186/1471-2105-14-52)
Supplement: Additional file 1 — Supplementary tables and figures. This pdf file contains Tables S1-S6 and Figures S1-S5. [file 1471-2105-14-52-S1.docx]

**Patricio Oyarzún, Jonathan J. Ellis, Mikael Bodén and Boštjan Kobe:**

**PREDIVAC: CD4+ T-cell epitope prediction for vaccine design that covers 95% of HLA class II DR protein diversity**

**Additional file 1**

**Supplementary Tables**

**Table S1 – Numbers of peptides in the PredivacDB database of high-affinity nonameric HLA class II ligands, indicating allele restriction and the respective contribution of IEDB, MHCBN and EPIMHC databases**

| **Allele** | **Peptides** | | | | | **PredivacDB** |
| --- | --- | --- | --- | --- | --- | --- |
|  | **IEDB** | **MHCBN** | **EPIMHC** | **Full list** | **Filtered list** | **Binding core** |
| DRB1*0101 | 919 | 120 | 52 | 1091 | 1007 | 815 |
| DRB1*0102 | 23 | 0 | 5 | 28 | 25 | 25 |
| DRB1*0301 | 170 | 19 | 21 | 210 | 202 | 180 |
| DRB1*0401 | 355 | 201 | 126 | 682 | 541 | 450 |
| DRB1*0402 | 37 | 21 | 43 | 101 | 82 | 59 |
| DRB1*0403 | 8 | 0 | 2 | 10 | 10 | 7 |
| DRB1*0404 | 94 | 18 | 52 | 164 | 143 | 102 |
| DRB1*0405 | 62 | 18 | 35 | 115 | 102 | 75 |
| DRB1*0406 | 0 | 3 | 2 | 5 | 8 | 8 |
| DRB1*0407 | 0 | 0 | 5 | 5 | 9 | 9 |
| DRB1*0701 | 189 | 11 | 34 | 234 | 219 | 192 |
| DRB1*0801 | 4 | 0 | 36 | 40 | 40 | 33 |
| DRB1*0802 | 39 | 3 | 2 | 44 | 40 | 35 |
| DRB1*0901 | 10 | 4 | 3 | 17 | 13 | 11 |
| DRB1*1101 | 167 | 64 | 25 | 256 | 238 | 226 |
| DRB1*1102 | 0 | 0 | 1 | 1 | 1 | 1 |
| DRB1*1103 | 1 | 0 | 0 | 1 | 1 | 1 |
| DRB1*1104 | 9 | 0 | 8 | 17 | 17 | 17 |
| DRB1*1201 | 3 | 0 | 9 | 12 | 12 | 9 |
| DRB1*1301 | 9 | 0 | 19 | 28 | 26 | 17 |
| DRB1*1302 | 81 | 4 | 18 | 103 | 91 | 73 |
| DRB1*1401 | 2 | 0 | 4 | 6 | 6 | 4 |
| DRB1*1501 | 227 | 33 | 19 | 279 | 225 | 172 |
| DRB1*1502 | 0 | 0 | 7 | 7 | 7 | 7 |
| DRB1*1601 | 1 | 0 | 0 | 1 | 1 | 1 |
| DRB3*0101 | 10 | 1 | 2 | 13 | 8 | 7 |
| DRB3*0301 | 1 | 0 | 0 | 1 | 1 | 1 |
| DRB4*0101 | 50 | 1 | 3 | 54 | 52 | 49 |
| DRB5*0101 | 143 | 13 | 9 | 165 | 140 | 109 |
| **Total** | **2614** | **534** | **542** | **3690** | **3267** | **2695** |

**Table S2 - Web-accessible methods for HLA class II binding prediction and CD4+ T cell epitope prediction employed in the benchmarking analysis**

| **Method** | **Prediction algorithm** | **Prediction scope** | **URL** |
| --- | --- | --- | --- |
| Predivac | SDR(specificity-determining residue) approach | Pan-specific | <http://predivac.biosci.uq.edu.au/> |
| NetMHCIIpan 2.0 | ANN (artificial neural network) | Pan-specific | http://www.cbs.dtu.dk/services/NetMHCIIpan/ |
| TEPITOPEpan | Virtual pockets | Pan-specific | http://www.biokdd.fudan.edu.cn/Service/TEPITOPEpan/TEPITOPEpan.html |
| MultiRTA | Thermodynamic principles | Pan-specific | http://www.bordnerlab.org/MultiRTA/ |
| IEDB (SMM-Align) | Matrix | Allele-specific | http://www.immuneepitope.org/ |
| IEDB (TEPITOPE) | Matrix | Allele-specific |  |
| IEDB (ARB) | Matrix | Allele-specific |  |
| SYFPEITHI | Motifs | Allele-specific | http://www.syfpeithi.de/ |
| Rankpep | Matrix | Allele-specific | http://imed.med.ucm.es/Tools/rankpep.html |
| MHC2Pred | SVM (support vector machine) | Allele-specific | http://www.imtech.res.in/raghava/mhc2pred/ |

**Table S3 - Predictive performance (AUC values) for CD4+ T cell epitope prediction of four pan-specific methods**

| **Allele** | **Epitopes** | **PREDIVAC** | **NetMHCIIPan** | **TEPITOPE** | **MultiRTA** |
| --- | --- | --- | --- | --- | --- |
| DRB1*0101 | 125 | 0.828 | 0.800 | 0.805 | 0.776 |
| DRB1*0102 | 4 | 0.860 | 0.869 | 0.846 | 0.828 |
| DRB1*0103 | 5 | 0.668 | 0.749 | 0.8 | 0.639 |
| DRB1*0301 | 173 | 0.658 | 0.693 | 0.643 | 0.657 |
| DRB1*0401 | 342 | 0.757 | 0.722 | 0.724 | 0.688 |
| DRB1*0402 | 33 | 0.574 | 0.589 | 0.616 | 0.551 |
| DRB1*0403 | 14 | 0.833 | 0.821 | 0.859 | 0.852 |
| DRB1*0404 | 46 | 0.762 | 0.718 | 0.747 | 0.712 |
| DRB1*0405 | 21 | 0.736 | 0.713 | 0.778 | 0.643 |
| DRB1*0406 | 6 | 0.775 | 0.684 | 0.815 | 0.845 |
| DRB1*0407 | 4 | 0.675 | 0.627 | 0.776 | 0.718 |
| DRB1*0408 | 2 | 0.963 | 0.997 | 0.956 | 0.987 |
| DRB1*0701 | 56 | 0.772 | 0.744 | 0.759 | 0.749 |
| DRB1*0703 | 1 | 1.000 | 0.864 | 0.914 | 0.717 |
| DRB1*0801 | 4 | 0.714 | 0.684 | 0.624 | 0.657 |
| DRB1*0802 | 2 | 0.929 | 0.695 | 0.85 | 0.735 |
| DRB1*0803 | 2 | 0.645 | 0.875 | 0.587 | 0.706 |
| DRB1*0901 | 13 | 0.701 | 0.669 | 0.745 | 0.642 |
| DRB1*1001 | 4 | 0.700 | 0.796 | 0.805 | 0.712 |
| DRB1*1101 | 88 | 0.710 | 0.78 | 0.733 | 0.688 |
| DRB1*1102 | 1 | 0.387 | 0.452 | 0.854 | 0.441 |
| DRB1*1103 | 3 | 0.337 | 0.518 | 0.317 | 0.492 |
| DRB1*1104 | 6 | 0.855 | 0.772 | 0.832 | 0.688 |
| DRB1*1201 | 3 | 0.911 | 0.961 | 0.887 | 0.87 |
| DRB1*1301 | 15 | 0.815 | 0.563 | 0.717 | 0.613 |
| DRB1*1302 | 10 | 0.721 | 0.772 | 0.749 | 0.764 |
| DRB1*1303 | 3 | 0.449 | 0.621 | 0.576 | 0.512 |
| DRB1*1401 | 16 | 0.561 | 0.734 | 0.719 | 0.687 |
| DRB1*1404 | 1 | 0.640 | 0.941 | 0.954 | 0.942 |
| DRB1*1405 | 2 | 0.670 | 0.896 | 0.819 | 0.923 |
| DRB1*1501 | 193 | 0.686 | 0.667 | 0.654 | 0.638 |
| DRB1*1502 | 20 | 0.709 | 0.707 | 0.661 | 0.615 |
| DRB1*1503 | 2 | 0.608 | 0.707 | 0.753 | 0.745 |
| DRB1*1601 | 5 | 0.715 | 0.650 | 0.549 | 0.734 |
| DRB1*1602 | 3 | 0.666 | 0.975 | 0.932 | 0.900 |
| DRB3*0101 | 12 | 0.850 | 0.808 | 0.838 | 0.890 |
| DRB3*0202 | 10 | 0.613 | 0.596 | 0.646 | 0.492 |
| DRB3*0301 | 1 | 0.962 | 0.957 | 0.911 | 0.953 |
| DRB4*0101 | 17 | 0.717 | 0.725 | 0.581 | 0.586 |
| DRB4*0103 | 1 | 0.856 | 0.996 | 0.99 | 0.99 |
| DRB5*0101 | 55 | 0.735 | 0.773 | 0.729 | 0.722 |
| DRB5*0102 | 1 | 0.924 | 0.994 | 0.885 | 0.758 |
|  |  |  |  |  |  |
| **Average** |  | **0.730** | **0.759** | **0.760** | **0.725** |

**Table S4 - MHC class II ligands with experimentally determined kinetic association rate constants (k_on_) obtained from the AntiJen database**

| **Epitope** | **Serotype** | **Species** | **k_on_ (M^-1^s^-1^)** |
| --- | --- | --- | --- |
| AERADLIAYLKAATAK | I-Ek | MOUSE | 47197.5 |
| AERADLIAYLKATAK | I-Ek | MOUSE | 54000 |
| AERADLIAYLKQATA | I-Ek | MOUSE | 56333.3 |
| AERADLIAYLKQATAK | I-Ek | MOUSE | 49110 |
| AERADLIAYLKQATK | I-Ek | MOUSE | 42845 |
| AERADLIAYLKQATKK | I-Ek | MOUSE | 44000 |
| ASQARPSQRHGSKYC | I-Ak | MOUSE | 1166.7 |
| AYLKQATK | I-Ek | MOUSE | 79000 |
| FAGIKKKANERADLIAYLKQATAK | I-Ek | MOUSE | 51.5 |
| FAGLKKANERADLIAYLKQATK | I-Ek | MOUSE | 42.5 |
| GAMNKALELFRKDIAAKYKELGYQG | I-Ek | MOUSE | 5.9 |
| GKKVITAFNDGLK | I-Ek | MOUSE | 58032.5 |
| IAGLGQGGK | I-Ek | MOUSE | 70000 |
| IAPLPQPPK | I-Ek | MOUSE | 83000 |
| IAYLKQA | I-Ek | MOUSE | 140000 |
| IAYLKQAT | I-Ek | MOUSE | 53667 |
| IAYLKQATK | I-Ek | MOUSE | 216000 |
| ISQAVHAAHAEINEAGR | I-Ad | MOUSE | 0.9 |
| KPSVQLRMATPLLLR | I-Ek | MOUSE | 400000 |
| KPSVQLRMATPLLMR | I-Ek | MOUSE | 188233.3 |
| KPSVQMRMATELLMR | I-Ek | MOUSE | 380000 |
| PKYVKQNTLKLATGM | HLA-DR1 | HUMAN | 1300 |
| QMRMATPLLMR | I-Ek | MOUSE | 270000 |
| VSRMRMATPLMQ | HLA-DR1 | HUMAN | 100 |
| YLKQATK | I-Ek | MOUSE | 143333.3 |
| YQAGFFLLTRILTIPQSLD | HLA-DR4 | HUMAN | 17750 |
|  |  |  |  |
| **Average** |  |  | **92121.9** |

**Table S5 - Dataset of immunodominant CD4+ T cell epitopes**

| **N** | **Pathogen** | **Protein antigen** | **ID** | **Length** | **Cluster** | **Epitope** | **Position** | **Restriction** | **References** |
| --- | --- | --- | --- | --- | --- | --- | --- | --- | --- |
| 1 | Influenza A virus (H3N2) | Hemagglutinin (HA) | gi\|94481520 | 566 |  | YVKQNTLKL | 324-332 | DRB1*0101 | [1-3] |
| 2 | Influenza A virus (H1N1) | M protein | gi\|238867319 | 252 |  | FVFTLTVPS | 62-70 | DRB1*0401 | [4] |
| 3 | *C. tetani* | Tetanus toxin (TT) | gi\|15022165 | 1310 |  | YIKANSKFI | 832-840 | DRB1*0101 | [5, 6] |
| 4 | *N. meningitidis* | Membrane protein Omp | gi\|89276847 | 381 |  | EFGTLRAGRVA | 106-116 | DRB1*0101 | [7] |
| 5 | Human herpesvirus 4 | Nuclear antigen  EBNA-1 | gi\|710394 | 203 |  | SLYNLRRGTAL | 78-88 | DRB1*0101 | [8] |
| 6 | Human herpesvirus 4 | BZLF1 protein | gi\|23893619 | 245 |  | QHYREVAAAKSSE | 198-210 | DRB1*0101 | [9] |
| 7 | Cytomegalovirus 5 | Envelope  glycoprotein B | gi\|330505 | 718 |  | DYSNTHSTRYV | 217-227 | DRB1*0701 | [10, 11] |
| 8 | Cytomegalovirus 5 | Envelope  Glycoprotein H | gi\|39842083 | 742 |  | HELLVLVKKAQL | 275-286 | DRB1*1101 | [10] |
| 9 | Dengue virus 1 | Polyprotein (NS3) | gi\|51850373 | 3392 |  | VIGLYGNGV | 1621-1629 | DRB1*1501 | [12, 13] |
| 10 | Dengue virus 3 | Polyprotein (NS3) | gi\|54401699 | 3390 |  | WITDFVGKTVW | 1824-1834 | DRB1*1501 | [12] |
| 11 | Dengue virus 4 | Polyprotein (capside) | gi\|12018170 | 3387 |  | FRKEIGRML | 83-91 | DRB1*0101 | [12, 14] |
| 12 | Hepatitis C virus | Polyprotein (NS3) | gi\|111283662 | 2939 |  | VAYYRGLDV | 1251-1259 | DRB1*1501 | [14] |
| 13 | Hepatitis C virus | Polyprotein (NS3) | gi\|111283662 | 2939 |  | GRHLIFCHSKR | 1388-1398 | DRB1*1501 | [14] |
| 14 | Hepatitis C virus | Polyprotein (NS3) | gi\|111283662 | 2939 |  | VLVLNPSVA | 1542-1550 | DRB1*1201 | [15, 16] |
| 15 | Hepatitis C virus | Polyprotein (NS3) | gi\|111283662 | 2939 |  | YMNTPGLPV | 1586-1594 | DRB1*0701 | [14, 16] |
| 16 | Hepatitis C virus | Polyprotein (NS3) | gi\|111283662 | 2939 |  | LVAYQATVC | 1694-1703 | DRB1*1501 | [14, 16] |
| 17 | Hepatitis C virus | Polyprotein (NS4) | gi\|111283662 | 2939 |  | IVPDREVLYR | 1775-1785 | DRB1*0301 | [14] |
| 18 | Hepatitis C virus | Polyprotein (NS4) | gi\|111283662 | 2939 |  | LAGLSTLPGNP | 1809-1817 | DRB1*1104 | [14, 16] |
| 19 | Hepatitis C virus | Polyprotein (NS4) | gi\|111283662 | 2939 |  | FNILGGWVA | 1879-1888 | DRB1*0101 | [14] |
| 20 | Hepatitis C virus | Polyprotein (NS4) | gi\|111283662 | 2939 |  | LVNLLPAILS | 1579-1587 | DRB1*0101 | [14] |
| 21 | HIV (HXB2 strain) | Gag (p17) | sp\|P04591 | 500 | ASRELERFAVNPGLL | RELERFAVNGLL | 39-49 | DRB1*1302 | [16, 17] |
| 22 | HIV (HXB2 strain) | Gag (p17) | sp\|P04591 | 500 | ASRELERFAVNPGLL | ERFAVNPGLL | 42-51 | DRB3*0301 | [17] |
| 23 | HIV (HXB2 strain) | Gag (p24) | sp\|P04591 | 500 | YVDRFYKTLRAEQASQEV | RFYKTLRAEQ | 300-308 | DRB1*1101 | [17, 18] |
| 24 | HIV (HXB2 strain) | Gag (p24) | sp\|P04591 | 500 | YVDRFYKTLRAEQASQEV | VDRFYKTLR | 298-306 | DRB1*1301 | [17, 18] |
| 25 | HIV (HXB2 strain) | Gag (p24) | sp\|P04591 | 500 | YVDRFYKTLRAEQASQEV | FYKTLRAEQAS | 301-311 | DRB1*1501 | [17, 18] |
| 26 | HIV (HXB2 strain) | Gag (p24) | sp\|P04591 | 500 | WIILGLNKIVRMYSPTSI | NKIVRMYSPTSI | 271-282 | DRB1*1101 | [17, 18] |
| 27 | HIV (HXB2 strain) | Gag (p24) | sp\|P04591 | 500 | WIILGLNKIVRMYSPTSI | ILGLNKIVRMY | 267-277 | DRB1*1301 | [17, 18] |
| 28 | HIV (HXB2 strain) | Gag (p24) | sp\|P04591 | 500 | WIILGLNKIVRMYSPTSI | WIILGLNKIVRM | 265-276 | DRB1*0101 | [17, 18] |
| 29 | HIV (HXB2 strain) | Gag (p24) | sp\|P04591 | 500 | WIILGLNKIVRMYSPTSI | KIVRMYSPTS | 272-281 | DRB1*0101 | [17-19] |
| 30 | HIV (HXB2 strain) | Gag (p24) | sp\|P04591 | 500 |  | EWDRVHPVHA | 211-220 | DRB1*0101 | [19] |
| 1 | HIV (HXB2 strain) | Gag (p24) | sp\|P04591 | 500 |  | PIVQNIQGQMV | 133-143 | DRB1*0101 | [19] |
| 32 | HIV (HXB2 strain) | Gag (p24) | sp\|P04591 | 500 |  | EVIPMFSALS | 167-176 | DRB1*0101 | [19] |
| 33 | *M. tuberculosis* | Acr antigen | gi\|21629888 | 86 | SEFAYGSFVRTVSLPVQAD | FAYGSFVRT | 66-74 | DRB1*0101 | [20] |
| 34 | *M. tuberculosis* | Acr antigen | gi\|21629888 | 86 | SEFAYGSFVRTVSLPVQAD | YGSFVRTVSL | 68-77 | DRB1*1501 | [20] |
| 35 | *Y. enterocolitica* | Hsp60 | gi\|139472697 | 211 |  | RVVINKDTTIII | 148-159 | DRB1*1302 | [21, 22] |
| 36 | *P. falciparum* | CSP | gi\|160161 | 411 | DIEKKIAKMEKASSVFNVVNS | KKIAKMEKASS | 381-391 | DRB1*1101 | [23, 24] |
| 37 | *P. falciparum* | CSP | gi\|160161 | 411 | DIEKKIAKMEKASSVFNVVNS | IAKMEKASSVFNV | 383-394 | DRB1*0401 | [23, 24] |
| 38 | *P. falciparum* | CSP | gi\|160161 | 411 | DIEKKIAKMEKASSVFNVVNS | MEKASSVFNV | 386-395 | DRB1*0901 | [23, 24] |
| 39 | *P. falciparum* | CSP | gi\|160161 | 411 | EYLNKIQNSLSTEWSPCSVT | KIQNSLSTEW | 337-346 | DRB1*0701 | [25] |
| 40 | *P. falciparum* | CSP | gi\|160161 | 411 | EYLNKIQNSLSTEWSPCSVT | YLNKIQNSLSTEW | 334-346 | DRB1*0401 | [26, 27] |
| 41 | *P. falciparum* | CSP | gi\|160161 | 411 | EYLNKIQNSLSTEWSPCSVT | LNKIQNSLSTEW | 335-346 | DRB1*0901 | [27] |
| 42 | *S. pneumoniae* | Protein kinase (stpk) | gi\|165932151 | 637 |  | FQISNYVGRKS | 416-426 | DRB1*1501 | [28] |

Minimal immunogenic regions in epitope clusters are underlined.

**Table S6 - Benchmarking analysis of immunodominant CD4+ T cell epitope prediction for four pan-specific methods**

| **N** | **Pathogen** | **Protein antigen** | **Predivac** | | **NetMHCIIPan** | | **TEPITOPEPan** | | **MultiRTA** | |
| --- | --- | --- | --- | --- | --- | --- | --- | --- | --- | --- |
|  |  |  | **Ranking** | **%** | **Ranking** | **%** | **Ranking** | **%** | **Ranking** | **%** |
| 1 | Influenza A virus (H3N2) | Hemagglutinin (HA) | 10 | 1.77 | 8 | 1.41 | 3 | 0.53 | 25 | 4.42 |
| 2 | Influenza A virus (H1N1) | M protein | 3 | 1.19 | 1 | 0.40 | 1 | 0.40 | 1 | 0.40 |
| 3 | *C. tetani* | Tetanus toxin (TT) | 16 | 1.22 | 32 | 2.44 | 19 | 1.45 | 74 | 5.65 |
| 4 | *N. meningitidis* | Membrane protein Omp | 2 | 0.52 | 1 | 0.26 | 6 | 1.57 | 1 | 0.26 |
| 5 | Human herpesvirus 4 | Nuclear antigen  EBNA-1 | 13 | 6.40 | 3 | 1.48 | 8 | 3.94 | 6 | 2.96 |
| 6 | Human herpesvirus 4 | BZLF1 protein | 2 | 0.82 | 2 | 0.82 | 3 | 1.22 | 1 | 0.41 |
| 7 | Cytomegalovirus 5 | Envelope  glycoprotein B | 6 | 0.84 | 38 | 5.29 | 22 | 3.06 | 6 | 0.84 |
| 8 | Cytomegalovirus 5 | Envelope  glycoprotein H | 3 | 0.40 | 8 | 1.08 | 5 | 0.67 | 55 | 7.41 |
| 9 | Dengue virus 1 | Polyprotein (NS3) | 20 | 0.59 | 45 | 1.33 | 89 | 2.62 | 6 | 0.18 |
| 10 | Dengue virus 3 | Polyprotein (NS3) | 8 | 0.24 | 618 | 18.23 | 325 | 9.59 | 394 | 11.62 |
| 11 | Dengue virus 4 | Polyprotein (capside) | 11 | 0.32 | 16 | 0.47 | 14 | 0.41 | 8 | 0.24 |
| 12 | Hepatitis C virus | Polyprotein (NS3) | 10 | 0.34 | 109 | 3.71 | 2 | 0.07 | 712 | 24.23 |
| 13 | Hepatitis C virus | Polyprotein (NS3) | 612 | 20.82 | 3 | 0.10 | 2015 | 68.56 | 153 | 5.21 |
| 14 | Hepatitis C virus | Polyprotein (NS3) | 24 | 0.82 | 80 | 2.72 | 138 | 4.70 | 75 | 2.55 |
| 15 | Hepatitis C virus | Polyprotein (NS3) | 10 | 0.34 | 55 | 1.87 | 5 | 0.17 | 19 | 0.65 |
| 16 | Hepatitis C virus | Polyprotein (NS3) | 97 | 3.30 | 257 | 8.74 | 21 | 0.71 | 955 | 32.49 |
| 17 | Hepatitis C virus | Polyprotein (NS4) | 7 | 0.24 | 3 | 0.10 | 36 | 1.22 | 5 | 0.17 |
| 18 | Hepatitis C virus | Polyprotein (NS4) | 44 | 1.50 | 1005 | 34.20 | 79 | 2.69 | 125 | 4.25 |
| 19 | Hepatitis C virus | Polyprotein (NS4) | 7 | 0.24 | 111 | 3.78 | 8 | 0.27 | 15 | 0.51 |
| 20 | Hepatitis C virus | Polyprotein (NS4) | 36 | 1.22 | 24 | 0.82 | 7 | 0.24 | 9 | 0.31 |
| 21 | HIV (HXB2 strain) | Gag (p17) | 61 | 12.20 | 190 | 38.00 | 466 | 93.20 | 362 | 72.40 |
| 22 | HIV (HXB2 strain) | Gag (p17) | 27 | 5.40 | 42 | 8.40 | 463 | 92.60 | 172 | 34.40 |
| 23 | HIV (HXB2 strain) | Gag (p24) | 12 | 2.40 | 4 | 0.80 | 11 | 2.20 | 89 | 17.80 |
| 24 | HIV (HXB2 strain) | Gag (p24) | 1 | 0.20 | 59 | 11.80 | 42 | 8.40 | 96 | 19.20 |
| 25 | HIV (HXB2 strain) | Gag (p24) | 14 | 2.80 | 41 | 8.20 | 78 | 15.60 | 39 | 7.80 |
| 26 | HIV (HXB2 strain) | Gag (p24) | 8 | 1.60 | 8 | 1.60 | 2 | 0.40 | 5 | 1.00 |
| 27 | HIV (HXB2 strain) | Gag (p24) | 5 | 1.00 | 4 | 0.80 | 6 | 1.20 | 1 | 0.20 |
| 28 | HIV (HXB2 strain) | Gag (p24) | 26 | 5.20 | 12 | 2.40 | 1 | 0.20 | 10 | 2.00 |
| 29 | HIV (HXB2 strain) | Gag (p24) | 4 | 0.80 | 1 | 0.20 | 3 | 0.60 | 3 | 0.60 |
| 30 | HIV (HXB2 strain) | Gag (p24) | 2 | 0.40 | 53 | 10.60 | 16 | 3.20 | 50 | 10.00 |
| 31 | HIV (HXB2 strain) | Gag (p24) | 3 | 0.60 | 15 | 3.00 | 5 | 1.00 | 16 | 3.20 |
| 32 | HIV (HXB2 strain) | Gag (p24) | 3 | 0.60 | 7 | 1.40 | 11 | 2.20 | 8 | 1.60 |
| 33 | *M. tuberculosis* | Acr antigen | 15 | 17.44 | 4 | 4.65 | 5 | 5.81 | 8 | 9.30 |
| 34 | *M. tuberculosis* | Acr antigen | 1 | 1.16 | 6 | 6.98 | 3 | 3.49 | 6 | 6.98 |
| 35 | *Y. enterocolitica* | Hsp60 | 6 | 2.84 | 1 | 0.47 | 6 | 2.84 | 6 | 2.84 |
| 36 | *P. falciparum* | CSP | 2 | 0.49 | 13 | 3.16 | 4 | 0.97 | 64 | 15.57 |
| 37 | *P. falciparum* | CSP | 9 | 2.19 | 18 | 4.38 | 31 | 7.54 | 30 | 7.30 |
| 38 | *P. falciparum* | CSP | 14 | 3.41 | 28 | 6.81 | 43 | 10.46 | 23 | 5.60 |
| 39 | *P. falciparum* | CSP | 16 | 3.89 | 36 | 8.76 | 8 | 1.95 | 11 | 2.68 |
| 40 | *P. falciparum* | CSP | 5 | 1.22 | 1 | 0.24 | 1 | 0.24 | 2 | 0.49 |
| 41 | *P. falciparum* | CSP | 27 | 6.57 | 15 | 3.65 | 15 | 3.65 | 22 | 5.35 |
| 42 | *S. pneumoniae* | Protein kinase (stpk) | 11 | 1.73 | 72 | 11.30 | 36 | 5.65 | 80 | 12.56 |
|  | | | |  |  |  |  |  |  |  |
| **Average** | | | | **2.79** |  | **5.40** |  | **8.75** |  | **8.18** |
| **Standard deviation** | | | | **4.38** |  | **8.09** |  | **22.08** |  | **13.29** |

The ranking corresponds, in a list sorted by the scores, to the place occupied by the immunodominant epitope score among the scores of all same-length peptides in the source protein, while the percentage is calculated from the ratio of the score divided by the total number of same-length peptides. The detailed information on the epitopes is presented in Table S2.

**Supplementary Figures**

**Figure S1 - SDR positions.**


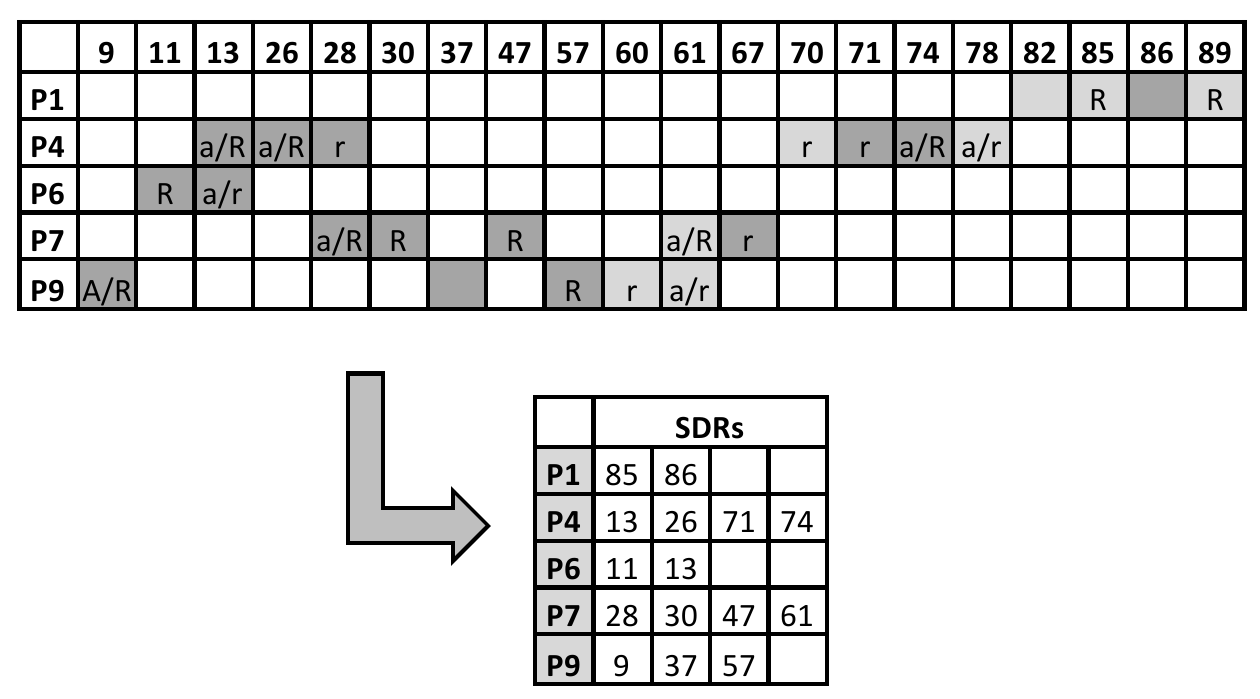


List of the SDR positions and criteria employed to select these positions in terms of polymorphism and calculated electrostatic effect on the binding groove. Columns correspond to the most conserved contacting positions identified in the MHC class II crystal structures analyzed. Rows are the peptide-binding positions (P1, P4, P6, P7 and P9). Cells in grey are highly polymorphic positions. In terms of electrostatic modelling, cells with letters A/a correspond to positions responsible for anchoring, while R/r corresponds to positions responsible for recognition. Uppercase corresponds to positions with a more significant effect [29, 30]. Dark grey cells are polymorphic positions and light grey cells are non-polymorphic positions. Although only polymorphic positions on the polymorphic β-domain were considered for SDR selection, the non-polymorphic position 85 was considered because it determines the size of the deepest cavity and the main anchoring site of the binding groove.

**Figure S2 - Cross-validation.**

Predivac cross-validation on HLA class II alleles having more than 25 associated peptide ligands in PredivacDB.

**Figure S3 - Predictive performance of four pan-specific methods in the identification of HLA class II DR1-restricted CD4+ T-cell epitopes of influenza virus.**

Predictive performance of Predivac, NetMHCIIPan, MultiRTA and TEPITOPEpan on DR1-restricted CD4+ T-cell epitopes from five infuenza virus (A/New Caledonia/20/1999(H1N1)) proteins: hemagglutinin (GenBank: AAP34324.1), neuraminidase (GenBank: ABW80984.1), nucleocapsid protein (GenBank: ACX46209.1), nonstructural protein 1 (GenBank: ACF41883.1) and the matrix protein 1 (GenBank: ACD37431.1).

**Figure S4 – Predictive performance of four pan-specific methods in the identification of HLA class II DR4-restricted CD4+ T-cell epitopes of influenza virus.**

Predictive performance of Predivac, NetMHCIIPan, MultiRTA and TEPITOPEpan on DR4-restricted CD4+ T-cell epitopes from five infuenza virus (A/New Caledonia/20/1999(H1N1)) proteins: hemagglutinin (GenBank: AAP34324.1), neuraminidase (GenBank: ABW80984.1), nucleocapsid protein (GenBank: ACX46209.1), nonstructural protein 1 (GenBank: ACF41883.1) and the matrix protein 1 (GenBank: ACD37431.1).

**Figure S5 – Predictive performance of four pan-specific methods in the high-specificity interval.**

Predictive performance of Predivac, NetMHCIIpan, TEPITOPEpan and MultiRTA in identifying immunodominant CD4+ T-cell epitopes. The fractional ROC curve corresponding to the specificity interval between 0.8-1.0 is shown.

**References**

1. Rothbard JB, Lechler RI, Howland K, Bal V, Eckels DD, Sekaly R, Long EO, Taylor WR, Lamb JR: **Structural model of HLA-DR1 restricted T cell antigen recognition**. *Cell* 1988, **52**(4):515-523.

2. Busch R, Strang G, Howland K, Rothbard JB: **Degenerate binding of immunogenic peptides to HLA-DR proteins on B cell surfaces**. *Int Immunol* 1990, **2**(5):443-451.

3. Stern LJ, Brown JH, Jardetzky TS, Gorga JC, Urban RG, Strominger JL, Wiley DC: **Crystal structure of the human class II MHC protein HLA-DR1 complexed with an influenza virus peptide**. *Nature* 1994, **368**(6468):215-221.

4. Linnemann T, Jung G, Walden P: **Detection and quantification of CD4(+) T cells with specificity for a new major histocompatibility complex class II-restricted influenza A virus matrix protein epitope in peripheral blood of influenza patients**. *J Virol* 2000, **74**(18):8740-8743.

5. Panina-Bordignon P, Tan A, Termijtelen A, Demotz S, Corradin G, Lanzavecchia A: **Universally immunogenic T cell epitopes: promiscuous binding to human MHC class II and promiscuous recognition by T cells**. *Eur J Immunol* 1989, **19**(12):2237-2242.

6. Diethelm-Okita BM, Raju R, Okita DK, Conti-Fine BM: **Epitope repertoire of human CD4+ T cells on tetanus toxin: identification of immunodominant sequence segments**. *J Infect Dis* 1997, **175**(2):382-391.

7. Meiring HD, Kuipers B, van Gaans-van den Brink JA, Poelen MC, Timmermans H, Baart G, Brugghe H, van Schie J, Boog CJ, de Jong AP *et al*: **Mass tag-assisted identification of naturally processed HLA class II-presented meningococcal peptides recognized by CD4+ T lymphocytes**. *J Immunol* 2005, **174**(9):5636-5643.

8. Khanna R, Burrows SR, Steigerwald-Mullen PM, Thomson SA, Kurilla MG, Moss DJ: **Isolation of cytotoxic T lymphocytes from healthy seropositive individuals specific for peptide epitopes from Epstein-Barr virus nuclear antigen 1: implications for viral persistence and tumor surveillance**. *Virology* 1995, **214**(2):633-637.

9. Stone JD, Demkowicz WE, Jr., Stern LJ: **HLA-restricted epitope identification and detection of functional T cell responses by using MHC-peptide and costimulatory microarrays**. *Proc Natl Acad Sci U S A* 2005, **102**(10):3744-3749.

10. Elkington R, Shoukry NH, Walker S, Crough T, Fazou C, Kaur A, Walker CM, Khanna R: **Cross-reactive recognition of human and primate cytomegalovirus sequences by human CD4 cytotoxic T lymphocytes specific for glycoprotein B and H**. *Eur J Immunol* 2004, **34**(11):3216-3226.

11. Crompton L, Khan N, Khanna R, Nayak L, Moss PA: **CD4+ T cells specific for glycoprotein B from cytomegalovirus exhibit extreme conservation of T-cell receptor usage between different individuals**. *Blood* 2008, **111**(4):2053-2061.

12. Zeng L, Kurane I, Okamoto Y, Ennis FA, Brinton MA: **Identification of amino acids involved in recognition by dengue virus NS3-specific, HLA-DR15-restricted cytotoxic CD4+ T-cell clones**. *J Virol* 1996, **70**(5):3108-3117.

13. Kurane I, Okamoto Y, Dai LC, Zeng LL, Brinton MA, Ennis FA: **Flavivirus-cross-reactive, HLA-DR15-restricted epitope on NS3 recognized by human CD4+ CD8- cytotoxic T lymphocyte clones**. *J Gen Virol* 1995, **76 ( Pt 9)**:2243-2249.

14. Gerlach JT, Ulsenheimer A, Gruner NH, Jung MC, Schraut W, Schirren CA, Heeg M, Scholz S, Witter K, Zahn R *et al*: **Minimal T-cell-stimulatory sequences and spectrum of HLA restriction of immunodominant CD4+ T-cell epitopes within hepatitis C virus NS3 and NS4 proteins**. *J Virol* 2005, **79**(19):12425-12433.

15. Diepolder HM, Gerlach JT, Zachoval R, Hoffmann RM, Jung MC, Wierenga EA, Scholz S, Santantonio T, Houghton M, Southwood S *et al*: **Immunodominant CD4+ T-cell epitope within nonstructural protein 3 in acute hepatitis C virus infection**. *J Virol* 1997, **71**(8):6011-6019.

16. Schulze zur Wiesch J, Lauer GM, Day CL, Kim AY, Ouchi K, Duncan JE, Wurcel AG, Timm J, Jones AM, Mothe B *et al*: **Broad repertoire of the CD4+ Th cell response in spontaneously controlled hepatitis C virus infection includes dominant and highly promiscuous epitopes**. *J Immunol* 2005, **175**(6):3603-3613.

17. Kaufmann DE, Bailey PM, Sidney J, Wagner B, Norris PJ, Johnston MN, Cosimi LA, Addo MM, Lichterfeld M, Altfeld M *et al*: **Comprehensive analysis of human immunodeficiency virus type 1-specific CD4 responses reveals marked immunodominance of gag and nef and the presence of broadly recognized peptides**. *J Virol* 2004, **78**(9):4463-4477.

18. Ramduth D, Day CL, Thobakgale CF, Mkhwanazi NP, de Pierres C, Reddy S, van der Stok M, Mncube Z, Nair K, Moodley ES *et al*: **Immunodominant HIV-1 Cd4+ T cell epitopes in chronic untreated clade C HIV-1 infection**. *PLoS One* 2009, **4**(4):e5013.

19. Boritz E, Palmer BE, Livingston B, Sette A, Wilson CC: **Diverse repertoire of HIV-1 p24-specific, IFN-gamma-producing CD4+ T cell clones following immune reconstitution on highly active antiretroviral therapy**. *J Immunol* 2003, **170**(2):1106-1116.

20. Caccamo N, Meraviglia S, La Mendola C, Bosze S, Hudecz F, Ivanyi J, Dieli F, Salerno A: **Characterization of HLA-DR- and TCR-binding residues of an immunodominant and genetically permissive peptide of the 16-kDa protein of Mycobacterium tuberculosis**. *Eur J Immunol* 2004, **34**(8):2220-2229.

21. Thiel A, Wu P, Lanowska M, Dong J, Radbruch A, Sieper J: **Identification of immunodominant CD4+ T cell epitopes in patients with Yersinia-induced reactive arthritis by cytometric cytokine secretion assay**. *Arthritis Rheum* 2006, **54**(11):3583-3590.

22. Mertz AK, Wu P, Sturniolo T, Stoll D, Rudwaleit M, Lauster R, Braun J, Sieper J: **Multispecific CD4+ T cell response to a single 12-mer epitope of the immunodominant heat-shock protein 60 of Yersinia enterocolitica in Yersinia-triggered reactive arthritis: overlap with the B27-restricted CD8 epitope, functional properties, and epitope presentation by multiple DR alleles**. *J Immunol* 2000, **164**(3):1529-1537.

23. Sinigaglia F, Guttinger M, Kilgus J, Doran DM, Matile H, Etlinger H, Trzeciak A, Gillessen D, Pink JR: **A malaria T-cell epitope recognized in association with most mouse and human MHC class II molecules**. *Nature* 1988, **336**(6201):778-780.

24. Romagnoli P, Takacs B, Kilgus J, Pink JR, Sinigaglia F: **Peptide-MHC interaction: a rational approach to vaccine design**. *Int Rev Immunol* 1990, **6**(1):61-73.

25. Moreno A, Clavijo P, Edelman R, Davis J, Sztein M, Herrington D, Nardin E: **Cytotoxic CD4+ T cells from a sporozoite-immunized volunteer recognize the Plasmodium falciparum CS protein**. *Int Immunol* 1991, **3**(10):997-1003.

26. Parra-Lopez C, Calvo-Calle JM, Cameron TO, Vargas LE, Salazar LM, Patarroyo ME, Nardin E, Stern LJ: **Major histocompatibility complex and T cell interactions of a universal T cell epitope from Plasmodium falciparum circumsporozoite protein**. *J Biol Chem* 2006, **281**(21):14907-14917.

27. Moreno A, Clavijo P, Edelman R, Davis J, Sztein M, Sinigaglia F, Nardin E: **CD4+ T cell clones obtained from Plasmodium falciparum sporozoite-immunized volunteers recognize polymorphic sequences of the circumsporozoite protein**. *J Immunol* 1993, **151**(1):489-499.

28. Aslam A, Mason A, Zemenides S, Chan H, Novakova L, Branny P, Finn A, Chapel H, Ogg GS: **Rapid effector function of circulating CD4+ T cells specific for immunodominant regions of the conserved serine/threonine kinase found in Streptococcus pneumoniae (StkP) in healthy adults**. *FEMS Immunol Med Microbiol* 2010, **60**(2):113-122.

29. Agudelo WA, Galindo JF, Ortiz M, Villaveces JL, Daza EE, Patarroyo ME: **Variations in the electrostatic landscape of class II human leukocyte antigen molecule induced by modifications in the myelin basic protein peptide: a theoretical approach**. *PLoS One* 2009, **4**(1):e4164.

30. Agudelo WA, Patarroyo ME: **Quantum chemical analysis of MHC-peptide interactions for vaccine design**. *Mini Rev Med Chem* 2010, **10**(8):746-758.
